# Frenectomy Managed by a Bilateral Pedicle Flap: A Case Report

**DOI:** 10.7759/cureus.71701

**Published:** 2024-10-17

**Authors:** Manju Krishnan, Ranjith Maari, Rudhra K, Anitha Balaji, BalaSubramaniam V

**Affiliations:** 1 Periodontics, Sree Balaji Dental College and Hospital, Chennai, IND

**Keywords:** double lateral pedicle, esthetic, frenectomy, maxillary labial frenum, single flap

## Abstract

In the oral cavity, the frenum is an anatomical structure composed of mucosal folds that connect the lip and cheek to the alveolar mucosa, gingiva, and underlying periosteum. An abnormally positioned maxillary labial frenum can contribute to the formation of a diastema and gingival recession. Various techniques have been proposed for the correction of aberrant frenal attachments, including frenectomy and frenal repositioning procedures. This case report presents an approach, combining frenectomy with a double lateral pedicle flap. The procedure involves closure across the midline by utilizing two lateral pedicle flaps, which are sutured together as a single flap before closure at the midline. This innovative technique ensures dual blood supply from both pedicles to the unified flap, promoting superior healing by primary intention, minimizing scar formation, and increasing the zone of the attached gingiva. In addition, the flap exhibits excellent color match with the adjacent tissue, contributing to aesthetically pleasing and functional outcomes.

## Introduction

The frenum is a fold of mucous membrane that connects the cheek and lip to the gingiva, periosteum, and alveolar mucosa. The maxillary labial frenum is specifically located at the junction between the inner surface of the upper lip and the midline between the central incisors [1]. Histological studies of the frenulum by Knox and Young revealed the presence of horizontal bands and oblique muscular fibers related to the orbicularis oris muscle [2,3]. However, studies by Henry, Levin, and Tsaknis identified thick collagenous and elastic fibers but found no muscle fibers in the frenum [4].

Placek et al. classified frenal attachments into four distinct types: (1) mucosal, where the frenum attaches to the mucogingival junction; (2) gingival, where the attachment extends into the attached gingiva; (3) papillary, where the attachment reaches the papilla; and (4) papillary-penetrating, where the frenum extends through the papilla and crosses onto the palatal aspect [5,6]. Clinically, the papillary and papillary-penetrating types often result in abnormalities that can be corrected through either frenectomy or frenotomy [5,6].

An aberrant maxillary labial frenum can act as an anatomic factor that promotes plaque accumulation and retention, potentially contributing to periodontal disease [5]. A frenectomy, which involves the complete surgical removal of the frenum and its underlying attachment to the bone, is often performed when an aberrant maxillary or mandibular frenum causes aesthetic concerns or functional issues [7]. Thickened or widened frenum attachments can lead to decreased vestibular depth, gingival recession, and the formation of a diastema between the central incisors. This enlarged frenum exerts abnormal tension on surrounding tissues, preventing proper tooth alignment and complicating oral hygiene efforts, which may further exacerbate oral health problems [8]. In such cases, frenectomy is recommended to remove or modify the frenum, improving both function and aesthetics [8,9].

This case report presents a novel surgical approach combining frenectomy with a double lateral pedicle flap technique. In this procedure, the two lateral pedicle flaps are sutured together as a single flap, allowing for blood supply from both pedicles. This dual blood supply promotes improved healing with minimal scarring, enhances gingival attachment, and yields superior aesthetic outcomes, ultimately benefiting the patient.

## Case presentation

A healthy 18-year-old patient presented with a chief complaint of bleeding gums in the upper front tooth region. Clinical examination revealed an aberrant maxillary labial frenum in the anterior region. The patient's medical history was non-contributory, and all relevant blood tests were within biological limits (Table 1).

**Table 1:** Hematology profile of the patient along with normal limits. All the values were under normal limits

Parameters	Results	Normal range
Hemoglobin	12.3g/dl	12-15 g/dl
Red blood cell count	4.36 million/cumm	3.5-4.5 million/cu mm
Total WBC count	7460 cells/cumm	4000-10000 cells/cumm
Neutrophils	73.30%	40-80%
Lymphocytes	20%	20-40%
Eosinophils	2.5%	1-6%
Basophils	0.2%	0-2%
Monocytes	2.1%	2-10%
Platelet counts	2.58 Lakhs/cumm	1.5-4.1 Lakhs/cumm
Erythrocyte sedimentation rate	9 mm/hr	5-20 mm/hr
Glucose fasting (FBS)	90.0mg/dL	74-100 mg/dL

**Figure 1:**
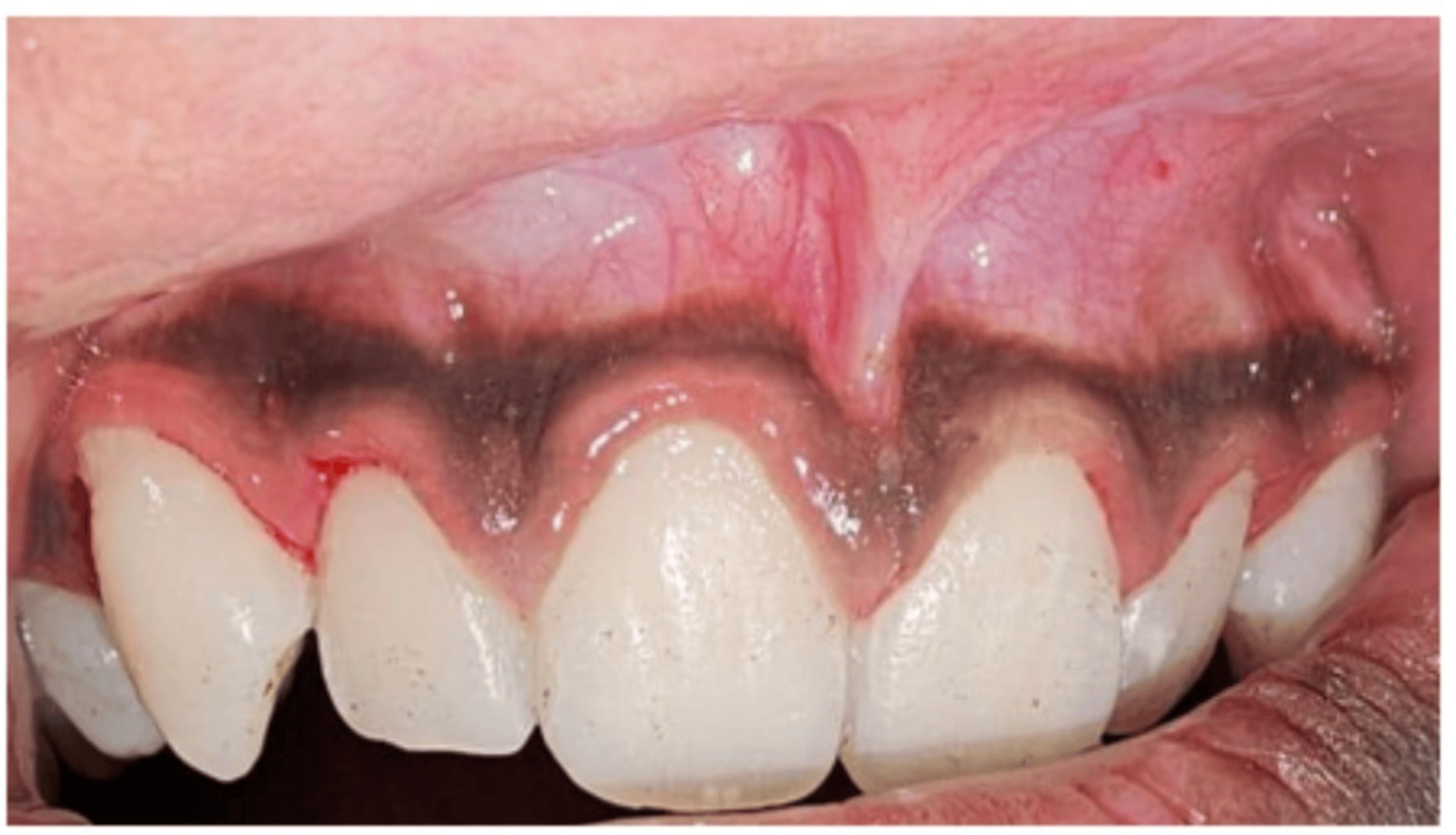
The frenum extending into the gingiva and interdental papilla, showing movement when tension is applied to the frenulum.

The patient exhibited good oral hygiene, with no trauma from occlusion. A hypertrophic frenal attachment was observed in the interdental area between the upper central incisors, and a positive blanch test was noted upon pulling the upper lip (Figure 1).

The blanch test during a frenectomy involves gently pulling the lip (for labial frenectomy) or tongue (for lingual frenectomy) to apply tension on the frenum while observing the surrounding tissue for blanching or whitening. This occurs due to reduced blood flow in areas under tension from the fibrous attachment. The surgeon uses this test to identify the extent of the frenum's attachment and its impact on nearby structures, guiding how much tissue needs to be released during the procedure to ensure proper movement without excess tissue removal.

The treatment plan included preoperative oral hygiene instructions, along with nonsurgical therapy to eliminate any predisposing or etiological factors contributing to gingivitis. Upon examination, the frenum was found to be fibrotic with a papillary attachment type. To reduce the extent of the surgical wound and minimize postoperative scarring, a novel frenectomy technique utilizing a double lateral pedicle flap was planned.

**Figure 2:**
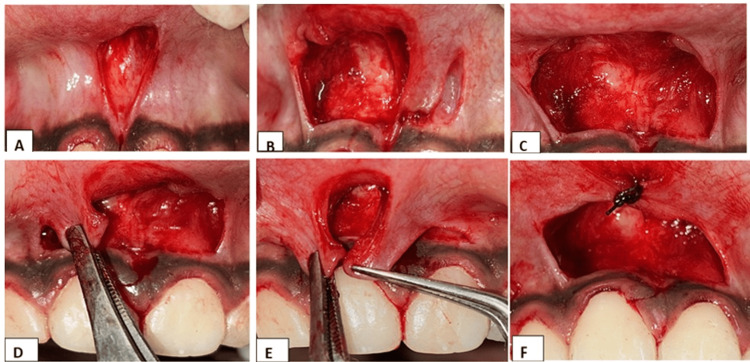
A V-shaped frenum outline was made (a). Blunt dissection relieved the fibrous attachment, creating a diamond shape (b). A 1 mm horizontal incision was placed at the gingival ends, followed by vertical incisions extending to the alveolar mucosa, resulting in a double lateral pedicle flap via split thickness (c–e). Both pedicle flaps were sutured together and approximated to the midline for primary closure (f).

The surgical procedure commenced with bilateral infiltration anesthesia at the lateral borders of the labial frenum. Using a #15 scalpel blade, primary incisions were made, and the frenum was engaged with a hemostat inserted into the vestibule. Incisions were placed along both the upper and undersurface of the hemostat until the frenum was fully released. This resulted in a V-shaped outline of the frenum (Figure 2a). Blunt dissection was performed down to the bone, releasing the fibrous attachment and creating a diamond-shaped wound (Figure 2b).

Horizontal incisions were then made 1 mm above the attached gingiva at both ends of the frenum, followed by vertical incisions extending into the alveolar mucosa (Figure 2c). This process formed double lateral pedicle flaps via split-thickness incisions (Figures 2d, 2e). The pedicle flaps were sutured together to create a single flap, which was then approximated and sutured at the midline to achieve primary closure (Figure 2f).

**Figure 3:**
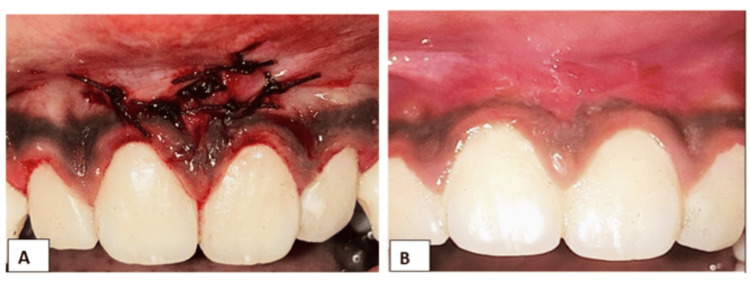
Complete suturing of the flap was performed (a). Two weeks postoperative image shows good healing without scar formation (b).

Complete suturing of the flap was performed (Figure 3a), and at the two-week postoperative follow-up, healing was excellent with no visible scar formation (Figure 3b).

A pedicle flap is a tissue that has a blood supply at the recipient site where it is harvested. The base of attachment will be in the recipient site itself, so with this benefit, the graft will soon integrate with the donor site area, since pedicles from either side, additional graft volume, and blood supply will be present. This novel frenectomy technique using a double lateral pedicle flap ensured optimal blood supply from both pedicles, promoting better healing by primary intention, preventing scar formation, and increasing the zone of the attached gingiva. The result was an aesthetically pleasing outcome and enhanced patient comfort.

## Discussion

Frenulum aberrations are one of the contributing factors to mucogingival abnormalities, which can compromise both aesthetics and functionality, ultimately affecting periodontal health. Numerous surgical modifications of frenectomy have been developed to enhance postoperative outcomes in various cases. Modern periodontal plastic surgery now focuses on precise and minimally invasive techniques to achieve more aesthetically pleasing and functional results [10]. In addition to achieving an aesthetically favorable appearance, maintaining an adequate zone of attached gingiva is essential for preventing recession, further underscoring the importance of frenulum removal in certain cases. The selection of flap design and suturing techniques in periodontal reconstructive surgery is crucial for ensuring optimal primary wound closure, which is a key biological requirement for successful periodontal reconstruction [11].

Friedman et al. emphasized the importance of attached gingiva, noting that inadequate attached gingiva allows muscles in the alveolar mucosa to pull the gingiva down, leading to gingival recession and bone loss due to plaque accumulation, which in turn hampers oral hygiene [5]. Similarly, Divater et al. highlighted the risk posed by frena that attach firmly to the gingival border, potentially causing gingival health issues due to muscle pull or plaque accumulation. The depth of the vestibular sulcus was also identified as a critical factor in gingival health [12].

The conventional frenectomy technique, introduced by Archer (1961) and Kruger (1964), involved scalpel excision of the frenum, which often resulted in the removal of interdental tissue, the palatine papilla, and the frenum itself. This exposed the underlying alveolar bone and often led to scarring [13]. To mitigate these complications, Miller introduced a procedure that combined frenectomy with a laterally placed pedicle flap, allowing full closure of the midline. This method provides primary intention healing, enhancing the formation of newly attached gingiva due to the lateral positioning of tissue with a preserved blood supply [14].

Hugund et al., in a series of cases, described that using a pedicle flap for frenectomy produced favorable aesthetic outcomes, with excellent color matching, increased attached gingiva, and no unaesthetic scarring due to healing by primary intention and prevention of coronal reformation [15]. In addition, Purushottam et al. emphasized the main advantage of the pedicle flap: its abundant blood supply from the well-nourished base, which facilitates vascular anastomoses at the healing site [16].

In this case report, a double lateral pedicle flap technique was employed as a reliable and predictable method for frenectomy. The primary advantage of this technique lies in its ability to provide an increased blood supply from both laterally repositioned pedicles, which promotes healing without scar formation. In addition, this technique offers a more substantial tissue supply for effective primary closure compared to a unilateral pedicle flap, further contributing to superior functional and aesthetic outcomes for the patient.

## Conclusions

This case introduces a novel and advanced technique in periodontal surgery by uniquely combining frenectomy and lateral pedicle flaps. While both methods are commonly employed, the innovation here lies in the way the two lateral pedicle flaps are sutured together to create a single unified flap that crosses the midline. This design ensures an enhanced blood supply from both pedicles, significantly improving vascularization at the surgical site. The increased blood flow promotes faster and more effective healing through primary intention, reducing complications such as delayed healing or scarring, which are more frequently seen with conventional frenectomy techniques. In addition, the dual-flap technique ensures better wound closure and prevents midline gapping, which can occur with traditional methods.

The use of double lateral pedicle flaps in this case also provides increased tissue bulk, contributing to a more natural appearance with superior color matching and an expanded zone of attached gingiva. This is essential for preventing future gingival recession and supporting long-term periodontal health. The enhanced tissue stability and functional integrity of this approach offer significant aesthetic and functional benefits compared to traditional frenectomy techniques or single pedicle flap designs. The combination of both techniques in this innovative manner represents a meaningful advancement in periodontal plastic surgery, offering a more effective solution for managing aberrant frena while maintaining optimal aesthetics and function.
